# Asymmetry, Abstraction, and Autonomy: Justifying Coarse-Graining in Statistical Mechanics

**DOI:** 10.1093/bjps/axy020

**Published:** 2018-06-26

**Authors:** Katie Robertson

## Abstract

While the fundamental laws of physics are time-reversal invariant, most macroscopic processes are irreversible. Given that the fundamental laws are taken to underpin all other processes, how can the fundamental time-symmetry be reconciled with the asymmetry manifest elsewhere? In statistical mechanics (SM), progress can be made with this question. What I dub the ‘Zwanzig–Zeh–Wallace framework’ can be used to construct the irreversible equations of SM from the underlying microdynamics. Yet this framework uses coarse-graining, a procedure that has faced much criticism. I focus on two objections in the literature: claims that coarse-graining makes time-asymmetry (i) ‘illusory’ and (ii) ‘anthropocentric’. I argue that these objections arise from an unsatisfactory justification of coarse-graining prevalent in the literature, rather than from coarse-graining itself. This justification relies on the idea of measurement imprecision. By considering the role that abstraction and autonomy play, I provide an alternative justification and offer replies to the illusory and anthropocentric objections. Finally, I consider the broader consequences of this alternative justification: the connection to debates about inter-theoretic reduction and the implication that the time-asymmetry in SM is weakly emergent.

1 Introduction
  1.1 Prospectus
2 The Zwanzig–Zeh–Wallace Framework
3 Why Does This Method Work?
  3.1 The special conditions account
  3.2 When is a density forwards-compatible?
4 Anthropocentrism and Illusion: Two Objections
  4.1 The two objections in more detail
  4.2 Against the justification by measurement imprecision
5 An Alternative Justification
  5.1 Abstraction and autonomy
  5.2 An illustration: the Game of Life
6 Reply to Illusory
7 Reply to Anthropocentric
8 The Wider Landscape: Concluding Remarks
  8.1 Inter-theoretic relations
  8.2 The nature of irreversibility

## 1 Introduction

Many processes occur in only one direction of time—people age, buildings crumble, eggs smash and gases spontaneously expand—towards the future. Rewinding a film of such processes displays an unphysical sequence of events: eggs cannot unsmash and people cannot become younger. A more technical way of describing the ‘directedness’ of such processes is to say that the laws governing these processes are not time-reversal invariant (TRI). That is, the time-reversal operator T does not send solutions of the equations—histories of the systems at issue—to solutions. (The time-reversal operator varies across theories, but here I take T to be the map t↦−t.)

In stark contrast, the laws of fundamental physics are TRI.^1^ The two sequences of events displayed by a film playing forwards, and in rewind, are both physical possibilities. That is, they are both solutions to the laws of fundamental physics. This leads to a traditional problem: given that the fundamental laws are taken to underpin all other processes, how can the fundamental time-symmetry be reconciled with the asymmetry manifest elsewhere?

It is not only the processes of our everyday experience that are irreversible. Many equations within physics are also irreversible. In particular, many equations in statistical physics are irreversible, such as the Boltzmann equation, the Langevin equation, the Pauli master equation […] the list goes on.

But within statistical mechanics (SM), much progress has been made with this traditional problem. The irreversible behaviour exhibited in non-equilibrium SM can be described by equations collectively called ‘master equations’, which give ‘a purposefully incomplete account of the conservative evolution of some underlying microscopic systems’ (Liu and Emch [2002], p. 479). This article focuses on one framework, originating in the work of Zwanzig ([1960]). The idea is that the irreversible equations of SM can be constructed from the reversible equations (of either classical or quantum mechanics (QM)). I will dub this the Zwanzig–Zeh–Wallace (ZZW) framework, since Zeh and Wallace are prominent later authors who have developed this framework.

However, this framework depends upon the procedure of coarse-graining, which has been heavily criticized. Redhead describes coarse-graining as ‘one of the most deceitful artifices I have ever come across in theoretical physics’ (Redhead [1996], p. 31; quoted in Uffink [2010], p. 197). Among the list of accusations against coarse-graining are: protests of empirical inadequacy, subjectivity, and incompatibility with scientific realism. So, if this construction method is to solve the puzzle of time-asymmetry in SM, a justification for coarse-graining is needed. The project of this article is to give such a justification.

### 1.1 Prospectus

I will answer two objections to coarse-graining in SM. In Section 2, I expound the ZZW framework and in Section 3, I consider why this framework works. Then I discuss two objections to coarse-graining, namely, that the asymmetry resulting from coarse-graining is illusory and/or anthropocentric. Section 4.1 outlines these two objections in detail. Section 4.2 describes the most prevalent—and I argue unsatisfactory—justification of coarse-graining in the literature, the measurement imprecision (MI) justification, which lies behind these objections. In Section 5, I outline my alternative justification of coarse-graining that can answer the two objections. These answers are given in Sections 6 and 7, respectively. In Section 8, I draw a broader consequence from this alternative justification: the coarse-grained asymmetry is weakly emergent.

## 2 The Zwanzig–Zeh–Wallace Framework

The ZZW framework provides a recipe for constructing irreversible dynamics from the underlying reversible dynamics. This framework works with both quantum and classical mechanics (CM) (Zwanzig [1961]), although I mainly discuss the classical case. It is clearest to see the framework as constructing an irreversible equation in three stages: First, move to the ensemble variant of the underlying microdynamics. Second, pick a coarse-graining projection $\hat{P}$, whose nature will be described below. Third, two moves are required to find an irreversible and autonomous equation for the coarse-grained probability density.

**Figure 1.**
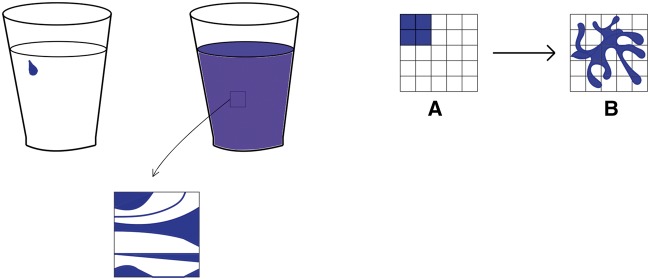
A drop of ink in a glass of water fibrillates throughout the whole volume, making the water look blue on a coarse-grained level (pictured on the left-hand side). Likewise, a probability density initially concentrated in one corner fibrillates across the available phase space (Sklar [1993]).

Stage 1: In classical SM, the state of an individual system is represented by a point in a phase space, *Γ*-space. (For *N* particles without internal degrees of freedom, *Γ*-space is 6*N*-dimensional). The system’s evolution is determined by Hamilton’s equations. However, there is also an ensemble variant of this description. Here probability densities over *Γ*-space, *ρ*, evolve according to Liouville’s equation, which, like Hamilton’s equations, is TRI.^2^

Stage 2: The concept of coarse-graining was originally introduced in a specific form by Gibbs ([1903]) which I first recall, before describing the generalized coarse-graining projections used by the ZZW framework.

Gibbs proposes that the accessible phase-space *Γ* is partitioned into small, finite volume elements $\Delta V_{m}$. The coarse-grained density $\rho_{cg}(q,p)$ is then defined by averaging the original probability density ρ(q,p) in each of these boxes. So coarse-graining throws away the information about how exactly the ensemble is distributed across each box.

Gibbs describes the evolution of the probability density by analogy with an ink drop. Dropping blue ink into a glass of water results in the whole glass appearing light blue. However, a drop of ink is an incompressible fluid and so its volume is constant. Upon examination under a microscope, we would see the drop of ink has just fibrillated into thin filaments across the whole glass; see Figure 1. So Gibbs’s idea is that like an incompressible fluid, *ρ* often fibrillates over the accessible phase space, as it evolves under the Liouvillean dynamics.

But because *ρ* behaves like an incompressible fluid, its volume is constant despite its fibrillation; and hence its Gibbs fine-grained entropy, $S_{fg}=-k_{B}\int_{\Gamma }\rho \ln\rho d^{3N}qd^{3N}p$ where *kB* denotes Boltzmann’s constant, is constant. Traditionally, this has been considered problematic, as the thermodynamic entropy increases. However, in a coarse-grained description, the density spreads smoothly throughout the available space, and this is well modelled by the coarse-grained probability density, *ρ_cg_*. This density has a different entropy, the Gibbs coarse-grained entropy,

(1) $$S_{cg}=-k_{B}\int_{\Gamma }\rho_{cg}\ln\rho_{cg}d^{3N}qd^{3N}p.$$

Unlike its fine-grained counterpart, *S_cg_* can increase.

Again, the ink analogy illuminates the discussion of time-evolution. From a macroscopic perspective, the ink smoothly spreads throughout the glass. In the SM case, this ‘smooth spreading’ of the coarse-grained density *ρ_cg_* is described by a ‘coarse-grained dynamics’, defined as follows: *ρ_cg_* evolves forward according to the usual Liouvillean dynamics for a small time interval Δt; and then it is coarse-grained; and this two-step process is iterated. This gives what Wallace ([unpublished]) terms the ‘coarse-grained forward (*C*^+^) dynamics’ (a label I henceforth adopt).

Note, however, that we could equally well have defined the coarse-grained backwards (*C*^*−*^) dynamics according to which *ρ_cg_* is evolved backwards for Δt by the Liouvillean dynamics, and then coarse-grained, then evolved backwards again, and so on. However, this *C*^*−*^ dynamics describes anti-thermodynamic trajectories (where entropy increases into the past) and so is ‘empirically disastrous’. The extent to which the success of the coarse-grained forwards, but not backwards, dynamics can be explained (in particular by appealing to cosmological considerations, such as postulating a ‘past hypothesis’) is controversial (see Albert [2000], Chapter 4; Earman [2006]; Wallace [unpublished]). But in this article, it will suffice to admit that the asymmetry has been added in here ‘by hand’ and thus that this project does not involve locating the ‘ultimate source’ of the time-asymmetry. For as announced in Section 1, I aim only to defend coarse-graining from various objections.

So far, I have only described Gibbs’s original coarse-graining. But in the ZZW framework, a more general notion of coarse-graining is used. A coarse-graining projection, $\hat{P}$, acts on the space of possible probability density functions.^3^ The important function of $\hat{P}$ is to split *ρ* into a ‘relevant’ part *ρ_r_* and an ‘irrelevant’ part *ρ_ir_*.

(2) $$\hat{P}\rho =:\rho_{r},(1-\hat{P})\rho =:\rho_{ir}\text{so}\text{that}\rho =\rho_{r}+\rho_{ir}.$$

Below are three examples of a coarse-graining projection $\hat{P}$ defining a relevant density *ρ_r_*. In these examples, the density is defined over a reduced number of degrees of freedom of the systems. Hence we speak of ‘relevant degrees of freedom’, as well as ‘relevant densities’.

1. The archetypal Gibbsian coarse-graining discussed above can be written as a projection, ${\hat{P}}_{cg}$. ${\hat{P}}_{cg}$ averages over small, finite volume elements $\Delta V_{m}\left(m=1,2\ldots \right)$ that cover the 6*N*-dimensional phase space *Γ*. These volume elements $\Delta V_{m}$ are sometimes referred to as ‘coarse-grained boxes’ or ‘cells of a partition’. (I write ‘$\Delta V_{m}$’ both for the region, and its volume.) Thus for $(q,p)\in \Delta V_{m}$, that is, the *m^th^* cell, we have
  (3) $${\hat{P}}_{cg}\rho (q,p):=\rho_{cg}(q,p):=\frac{1}{\Delta V_{m}}\int_{\Delta V_{m}}\rho (q\prime ,p\prime )dq\prime dp\prime =:\frac{\rho_{m}}{\Delta V_{m}},$$
  so that for a general (*q*, *p*) we sum over the cells with characteristic functions
  (4) $${\hat{P}}_{cg}\rho (q,p):=\rho_{cg}(q,p):=\sum_{m}\chi_{\Delta V_{m}}(q,p).\frac{\rho_{m}}{\Delta V_{m}}$$
  The action of ${\hat{P}}_{cg}$ is to smooth the density *ρ* to be uniform across each box, while leaving the probability of being in any single box invariant; for all *m*, $\int_{\Delta V_{m}}{\hat{P}}_{cg}\rho =\int_{\Delta V_{m}}\rho$.
2. Correlations between particles are discarded by appropriate integration, that is, by taking a marginal distribution. And this can be thought of as applying a projection ${\hat{P}}_{\mu }$. This projection takes you from a probability density on the full phase space, *Γ*-space (6*N*-dimensional for *N* point particles), to the one-particle marginal density, which describes the probability that particle *i* will be at a particular point in (six-dimensional) *μ*-space, that is, have a given $(\vec{q},\vec{p})\in {\mathbb{R}}^{6}$.
  Thus, the mapping from *Γ*-space densities to *μ*-space densities destroys information about the correlations between different particles and cannot be inverted.
  In the BBGKY hierarchy we define a system of correlation functions, where *f_s_* gives the probability that *s* particles have a given position and momenta. Generally, the evolution of *f_s_* depends on $f_{s+1}$, and $f_{s+1}$ depends on $f_{s+2}\ldots$ all the way to *f_N_* (where *N* is the totally number of particles). But—under certain physical conditions—this chain of equations can be truncated at a given point, that is, all correlations beyond the three-particle correlations can be thrown away (Huang [1987], p. 65).
  A projection akin to ${\hat{P}}_{\mu }$ is used in constructing the Boltzmann equation (see Wallace [2015], p. 292; for an explicit construction of the Prigogine–Brout equation—a cousin of the Boltzmann equation—see Zwanzig [1960], p. 1340).
3. The diagonalization projection ${\hat{P}}_{dia}$ applies to quantum systems and removes off-diagonal elements of the density matrix (with respect to some chosen basis). This partitioning into diagonal and off-diagonal matrix elements (relevant and irrelevant, respectively) is used in the derivation of the Pauli master equation (Zwanzig [1960], p. 1339), where discarding the off-diagonal elements amounts to ignoring interference terms.

Given a coarse-graining projection $\hat{P}$, the next aim is to find an equation for just the relevant degrees of freedom described by *ρ_r_*. By re-arranging the Liouville equation in terms of the two densities, *ρ_r_* and *ρ_ir_*, we find the pre-master equation (for the steps to the pre-master equation, see Zwanzig [1960], Section 2):

(5) $$\frac{\partial \rho_{r}(t)}{\partial t}=\hat{F}\rho_{ir}(t_{0})+\int_{t_{0}}^{t}dt\prime \hat{G}(t\prime )\rho_{r}(t-t\prime ),$$

where $\hat{F}:=\hat{P}Le^{-it(1-\hat{P})L}$ and $\hat{G}(t\prime ):=\hat{P}Le^{it\prime (1-\hat{P})L}(1-\hat{P})L$. *L* represents the Liouvillean evolution.

This pre-master equation is formally exact and so the time-reversibility remains. The first term on the right-hand side depends on the irrelevant degrees of freedom, *ρ_ir_*. The second term is non-Markovian; the evolution of *ρ_r_* at *t* depends on the history of the system between *t*_0_ and *t* as evidenced by the integral between $t\prime =t_{0}$ and *t*. This is unlike classical mechanical trajectories for which, given the current state, the future evolution is determined without any information about the system’s history.

Stage 3: Next, two assumptions are used to arrive at an autonomous and irreversible equation for the relevant degrees of freedom. ‘Autonomy’ requires that the dynamical evolution of *ρ_r_* has no explicit dependence on *ρ_ir_* or *t*.^4^ The reversible pre-master Equation (5) is of the form $\frac{\partial \rho_{r}(t)}{\partial t}=f(\rho_{r}(t),\rho_{ir}(t),t)$ and so is not a time-independent or autonomous equation.

In general, an autonomous dynamics for *ρ_r_* is in no way guaranteed; since *ρ* can be decomposed any way we like, the aspects of *ρ* we have dubbed ‘relevant’ (*ρ_r_*) need not be dynamically autonomous or independent from the irrelevant aspects. Two steps are required:

1. The initial state assumption states that the first term vanishes. This is achieved by stipulating that $\rho_{ir}(t_{0})=0$.^5^ When $\rho_{ir}(t_{0})=0$, Equation (5) becomes a closed equation for $\rho_{r}(t)$.
2. The Markovian approximation requires that $\hat{G}(t\prime )$ decreases to zero over a certain timescale, the ‘relaxation time’, *τ*. Thus, for times t′ greater than the relaxation time *τ*, $\hat{G}(t\prime )=0$. Furthermore, it requires that *ρ_r_* does not vary much over this timescale *τ*, and so $\hat{G}(t\prime )$ drops off more rapidly than the timescales over which *ρ_r_* evolves. To sum up: the key physical idea of the Markovian approximation is that there is a relaxation time *τ* over which the integral kernel drops off and over which *ρ_r_* does not vary much (Wallace [2015], p. 292).^6^

Provided that these physical features hold, then the following mathematical moves can be made:

- If the integral upper limit *t* is greater than *τ* extending the integration interval to ∞ makes no difference to the value of the integral; $\int_{t_{0}}^{\infty }dt\prime \hat{G}(t\prime )\rho_{r}(t-t\prime )≃\int_{t_{0}}^{t}dt\prime \hat{G}(t\prime )\rho_{r}(t-t\prime )$.
- If *ρ_r_* varies very slowly over *τ*, $\rho_{r}(t-t\prime )\approx \rho_{r}(t)$ for t′<τ. (If t′>τ this approximation does not hold, but since $\rho_{r}(t-t\prime )$ is multiplied by $\hat{G}(t\prime )$ which is ≈0 for t′>τ, we can replace $\rho_{r}(t-t\prime )$ by $\rho_{r}(t)$.)
- Thus, if the Markovian approximation holds, we can replace the second term $\int_{t_{0}}^{t}dt\prime \hat{G}(t\prime )\rho_{r}(t-t\prime )$ of Equation (5) by $\int_{t_{0}}^{\infty }dt\prime \hat{G}(t\prime )\rho_{r}(t)$.

Provided that the initial state assumption and the Markovian approximation hold, we thus arrive at an autonomous equation—the master equation—for the relevant degrees of freedom, *ρ_r_*:

(6) $$\frac{\partial \rho_{r}(t)}{\partial t}\approx \hat{D}\rho_{r}(t),$$

where $\hat{D}:=\int_{t_{0}}^{\infty }dt\prime \hat{G}(t\prime )$.

This completes Stage 3.

For our purposes, there are three comments to make. First, the schematic Equation (6) has specific forms for specific systems (Penrose [1979], p. 1986); ‘various particular cases of it include the (empirically verified) equations of decoherence, of radioactive decay, and of diffusion and equilibration in dilute gases’ (Wallace [2015], p. 292).

Second, we can now describe the irreversible behaviour using a generalized version of the Gibbs coarse-grained entropy. The coarse-grained Gibbs entropy *S_cg_* (in Equation (1)) can be written as a functional of *ρ* and ${\hat{P}}_{cg}$:

(7) $$S_{cg}[\hat{P};\rho ]=-k_{B}\int {\hat{P}}_{cg}\rho (q,p)\ln{\hat{P}}_{cg}\rho (q,p)d^{3N}qd^{3N}p.$$

And similarly more generally: we define, for any ZZW projection $\hat{P}$, obeying Equations (5) and (6), the entropy:

(8) $$S[\rho_{r}]:=S[\hat{P};\rho ]:=-k_{B}\int \hat{P}\rho (q,p)\ln\hat{P}\rho (q,p)d^{3N}qd^{3N}p.$$

 This quantity can increase—like *S_cg_*, as noted after Equation (1). Thus Zeh ([2007], p. 65) writes: ‘if $\hat{P}$ only destroys information, the master equation describes never-decreasing entropy’:

(9) $$\frac{dS[\rho_{r}]}{dt}\geq 0.$$

For a proof, see (Tolman [1938], p. 171; Huang [1987], p. 74; Reif [2009], p. 624; and for the quantum context, see Landsberg [1990], p. 145).

Finally, and most importantly for our interests: this closed Equation (6) is irreversible (Zwanzig [1960], p. 1340).

## 3 Why Does This Method Work?

Why does the ZZW framework lead to empirically successful equations? This success is surprising because, after all, the coarse-graining projection (and the ensuing *C*^+^ dynamics) cannot be implemented by the ‘official’ microdynamics. Given Liouville’s theorem, the microdynamics of the closed system cannot really cause the velocity correlations to be erased (in the case of the Boltzmann equation), or really delete the off-diagonal density matrix elements (in the case of the Pauli master equation). In short: the TRI microdynamics of the closed system cannot dynamically implement the coarse-graining projection.

In order to explain the success of irreversible equations in SM there have been three broad strategies:

(1) Interventionists (for example, Bergmann and Lebowitz [1955]; Blatt [1959]; Ridderbos and Redhead [1998]) argue that perturbations from the environment cannot be neglected. Thus, the system cannot be treated as closed. (In the ZZW terminology, the environment dynamically implements the projection, so that *ρ_r_*, rather than *ρ*, is the correct description of the subsystem.)
(2) Others advocate changing the underlying microdynamics so that the coarse-graining projection is dynamically implemented. Albert ([2000]) and Prigogine and Stengers ([1984]) advocate non-TRI microdynamics in the quantum and classical case, respectively. (In the ZZW terminology, the non-TRI dynamics yields $\rho \mapsto \rho_{r}$.)
(3) Some, such as Wallace ([2012]), propose that under special conditions the irreversible SM dynamics will give the same density over the relevant degrees of freedom as the microdynamics.

For the remainder of the article, I only focus on the third of these strategies, which I call ‘the special conditions’ account. In Section 3.1, I consider this account and the required ‘meshing’ condition. Section 3.2 considers when a density satisfies this condition and reports Wallace’s proposal. This will lead into the idea of a past hypothesis; (although, as mentioned in Stage 2 of Section 2, an in-depth discussion of the controversial past hypothesis is beyond the scope of this article).

### 3.1 The special conditions account

The third strategy claims that under certain conditions the microdynamics will induce the same probabilities for the relevant degrees of freedom, as the *C*^+^ coarse-grained dynamics governing *ρ_r_*. On this view, the generalized coarse-graining projection is not dynamically implemented. Thus, *ρ* and *ρ_r_* are two distinct densities.

**Figure 2.**
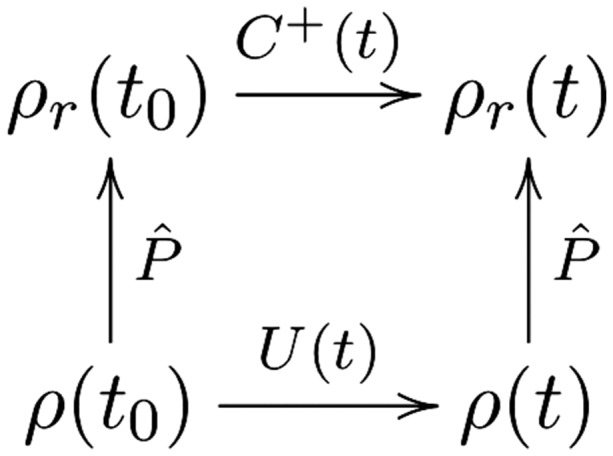
*ρ* and $\hat{P}$ are forwards-compatible if the two routes to $\rho_{r}(t)$ give the same answer.

How do we find *ρ_r_* at a given time *T*? There are two ‘routes’. As discussed in Section 2, the *C*^+^ dynamics for a period $t_{0}<t<T$ is defined by evolving the density by the microdynamics $\hat{U}$ for a very short time Δt, then applying the projection $\hat{P}$, then evolving under $\hat{U}$ for Δt, then $\hat{P}\ldots$ and so on. This means that irrelevant details are thrown away at every step. In contrast, the Liouvillean microdynamics $\hat{U}$ evolves the full density *ρ* for the period $t_{0}<t<T$; and then one finds the relevant part of the density by applying $\hat{P}$ at *T*; so on this ‘route’, coarse-graining occurs only once at the end of the time-period. Thus, the condition that these two different dynamics give the same density $\rho_{r}(T)$ can be expressed by the diagram in Figure 2 commuting.

Following the terminology suggested by Wallace ([unpublished]), let us call those states *ρ* for which the diagram in Figure 2 commutes ‘forwards compatible’ with coarse-graining $\hat{P}$. So forwards compatibility means that it does not matter whether you coarse-grain at every time step Δt or just once, at the end. Note that forwards compatibility is relative to a particular choice of coarse-graining $\hat{P}$. Thus this is a condition of ‘harmony’ between the evolution of *ρ* and the coarse-graining $\hat{P}$. For example, had the size of the coarse-graining boxes $\Delta V_{m}$ averaged over in Gibbs’s original example been chosen to be very large, then *ρ* might well not be forwards-compatible with this coarse-graining, ${\hat{P}}_{cg}$. In the wider literature on inter-theoretic relations, such a forwards-compatible scenario is sometimes described as ‘meshing’ dynamics (for example, Butterfield [2012]; List [unpublished]).

However, we cannot expect harmony to ‘reign supreme’. Not all densities *ρ* will satisfy Figure 2’s meshing condition: Loschmidt’s reversibility objection vividly reminds us that if we were to reverse the momenta of the components of a fibrillating ink drop, it would coalesce back in a manner incompatible with the ‘smooth-spreading out’ coarse-grained dynamics. (More specifically, the time-reverse of a density *ρ* initially forwards-compatible and on a trajectory of increasing entropy will not itself be forwards-compatible.)

And due to Poincaré’s recurrence theorem, nor will any *ρ* satisfy the meshing condition for all time. (More specifically, in the ZZW framework, recurrence implies that the integral kernel $\hat{G}$ in Equation (5) must increase again so that at the recurrence time it has returned to its original value. Therefore the upper limit of the integral in the Markovian approximation strictly cannot be taken to ∞, but at most to some large—but sub-recurrent—time *T*. Consequently, the Markovian approximation is only valid for sub-recurrent times.)

### 3.2 When is a density forwards-compatible?

Characterizing those densities *ρ* that are forwards-compatible is a harder job than ruling out candidate densities. A density *ρ* will be forwards-compatible provided that the density *ρ_ir_* (and the details such as correlations encoded in it) that are thrown away by $\hat{P}$ do not matter for the forwards-evolution of *ρ_r_*. One clear case where this is not true is Hahn’s ([1950]) spin-echo experiment. The application of a radio-frequency pulse causes dephased spins (precessing in a magnetic field) to realign and thus emit an ‘echo’ signal (for a recent philosophical discussion, see Frigg [2010], Section 3.5.1). The correlations—that are ignored from the coarse-graining perspective—are crucial for the later ‘echo signal’. Indeed, the spin-echo experiment has been described as a ‘Loschmidt demon’ that reverses the velocities v↦−v.^7^

Given the above discussion of the Loschmidt reversibility objection, here too the density *ρ* is clearly not forwards-compatible. Consequently, the spin-echo is not a surprising counterexample to the coarse-graining framework—which we can only expect to be successful when Figure 2 commutes, that is, when the information (in this case, correlations) thrown away by the coarse-graining projection $\hat{P}$ are not crucial—unlike the spin-echo case.

Ridderbos and Redhead ([1998], p. 1237) and Blatt ([1959], p. 749) generalize from the spin-echo case to reject the coarse-graining framework altogether.^8^ However, rather than claiming that the spin-echo case reveals coarse-graining to be empirically inadequate, it seems fairer to say the density *ρ* is patently not forwards-compatible and so we do not expect coarse-graining methods to apply.^9^

Naturally, the following question arises: why should we expect the spin-echo (‘correlations-are-crucial’) type of case to be the exception rather than the rule? To this, the reply can only be that ‘nature is kind’: often—that is, in the irreversible equations of SM—*ρ_ir_* is irrelevant for the evolution of *ρ_r_*.

Nonetheless, one might ask what informative condition can be used to pick out the forwards-compatible scenarios. Since the presence of ‘crucial correlations’ was the problem in the spin-echo case, perhaps removing them is the answer, that is, ensuring there is no irrelevant information at all is one way to avoid the failure of compatibility. Indeed, this is what the initial state assumption $\rho_{ir}(t_{0})=0$ in Section 2 achieved—and alongside the Markovian approximation, this was used to construct the *C*^+^ dynamics. In similar vein, Wallace ([unpublished], p. 19) stipulates that ‘simple’ initial densities *ρ* will not have crucial conspiratorial correlations encoded in their irrelevant degrees of freedom; he defines ‘a simple distribution as any distribution specifiable in a closed form in a simple way without specifying it as the time evolution of some other distribution’.^10^

Note, however, that such a condition—the initial state assumption or Wallace’s simplicity condition—can only be applied once.^11^ The initial state A in Figure 1—confined to four Gibbsian cells, or, in the analogy, the ink drop’s initial state—is simple (or equivalently it satisfies the ZZW initial state assumption). However, it then fibrillates over the available phase space and thus is no longer simple. While initially at *t*_0_ there was no irrelevant information, this is no longer the case: $\rho_{ir}(t_{1})\neq 0$. Yet—we hope!—$\rho (t_{1})$ is still forwards-compatible. Accordingly, the simple states are a subset of the forwards-compatible states. Thus, given the microdynamics, imposing such an initial condition is a sufficient but not necessary initial condition for ensuring that *ρ* is forwards-compatible.^12^

Given that such an initial condition can only be applied once, when should we apply it? Practising physicists apply it at the beginning of the time of interest, *t*_0_ (option 1). But this leads to a problem akin to that facing Boltzmann’s combinatoric argument. By parity of reasoning, this licences the construction of the *C^−^* dynamics prior to *t*_0_, and the *C^−^* dynamics yields anti-thermodynamic trajectories prior to *t*_0_.

Such parity problems motivate the past hypothesis; in the Boltzmannian case that the initial macrostate of the universe had a ‘low entropy’ (Albert [2000], Chapter 4). Here, this parity problem motivates Wallace ([unpublished], p. 22) to apply the initial state assumption to the beginning of the universe (option 2). An in-depth analysis of the past hypothesis—and the different possible forms it could take; see (Wallace [unpublished])—is not possible here, but I can allay one worry. Provided Markovian approximation holds good, the choice between applying this condition in the manner of physicists (Option 1) and a past hypothesis (Option 2) will not lead to dramatic empirical differences.^13^

In summary, when the forwards-compatibility condition fulfilled, *C*^+^ dynamics gives the same values for relevant *ρ_r_* as the microdynamics. Not all densities *ρ* are forwards-compatible and nor is any density forwards-compatible for all times, as shown by the reversibility and recurrence objections, respectively. When considering how to determine whether a given *ρ* is forwards-compatible or not, one suggestion was that a probability density will be forwards-compatible if it satisfies the initial state assumption at *t*_0_ (or in Wallace’s terminology is simple at *t*_0_). However, whether *t*_0_ should be taken to be at the beginning of time of interest (Option 1) or the beginning of the universe (Option 2) is a contentious matter.

## 4 Anthropocentrism and Illusion: Two Objections

If coarse-graining is empirically successful (as I have claimed) then perhaps no further justification is required. This would be a tempting line to take, were it not for the literature’s containing a barrage of criticisms of coarse-graining. For example, coarse-graining ‘seems repugnant to many authors’ (Uffink [2010], p. 197) and is even claimed to be ‘deceitful’ (Redhead [1996], p. 31). The coarse-grained time-asymmetry is also called ‘illusory’ (Prigogine [1980]) and potentially ‘subjective’ (Denbigh and Denbigh [1985], p. 53).

This purported subjectivity of coarse-graining leads to concerns about the status of the time-asymmetry. According to Davies ([1977], p. 77), ‘it is indeed a matter of philosophy rather than physics to decide if the coarse-grained asymmetry is ‘real’ or not’. Furthermore, the potentially unusual or subjective status of the coarse-grained asymmetry within physics leads Grünbaum ([1973]) to discuss whether scientific realism is incompatible with coarse-graining approaches in SM. More broadly, determining this status of the asymmetry is part of a wider philosophical project of untangling ‘what is genuinely an aspect of reality from what is a kind of appearance, or artifact, of the particular perspective from which we regard reality’ (Price [1996], p. 4).

Summing up, it seems to me that these objections can be divided into two camps:

Illusory: First, the asymmetry is a mere artefact of coarse-graining and so is illusory.

Anthropocentric: Second, it arises from our perspective and so is anthropocentric.

Given these concerns and objections, coarse-graining requires some conceptual, not just empirical, justification.

I propose that this task can be split into two:

Choice: What is the justification for the choice of coarse-graining projection?

At All: Why is it legitimate to coarse-grain at all?

A justification for coarse-graining may of course purport to answer both questions. And the answers may be linked. For example, if the justification for the choice of coarse-graining projection was deemed to be unacceptably subjective, then this might lead one to believe that coarse-graining at all is unacceptable. However, the two issues can also come apart. For example, a justification for coarse-graining might only motivate why it is an acceptable procedure in general, but remain silent on how to choose a particular coarse-graining projection.

In Section 4.2, I will consider and reject the ‘measurement imprecision’ justification and discuss how it lies behind the illusory and anthropocentric objections, followed, in Section 5, by my favoured justification. But first, I consider the two objections in more detail—in Section 4.1.

### 4.1 The two objections in more detail

The claim that the coarse-grained asymmetry is an ‘illusion’ (Prigogine [1980]; as cited in Denbigh and Denbigh [1985], p. 56) is rooted in the action of $\hat{P}$. The contention is that $\hat{P}$ ‘distorts’ *ρ* and the gap between *ρ* and *ρ_r_* is the source of the coarse-grained asymmetry. Every time we apply $\hat{P}$ we edge away from the correct density *ρ*—in particular, we edge away from the correct value of the Gibbs (fine-grained) entropy by a certain amount: ‘the required increase in the coarse-grained entropy is obtained by disregarding the dynamical constraints on the system’ (Ridderbos, p. 66). By repeatedly coarse-graining (as is done in the *C*^+^ dynamics), we generate the coarse-grained asymmetry: ‘The repeated coarse-graining operators appear to be added “by hand”, in deviation from the true dynamical evolution provided by *U_t_*’ (Uffink [2010], p. 197). That is, the coarse-grained asymmetry exists merely in virtue of the continual coarse-graining in the *C*^+^ dynamics—each coarse-graining increases *S_cg_* by some small amount so that eventually an asymmetry is produced: ‘Perhaps most worrying, the irreversible behaviour of *S_cg_* arises almost solely due to the coarse-graining’ (Callender [1999], p. 360). Thus, since the asymmetry stems from the infidelity of coarse-graining, it is illusory.

This illusory objection has the following form:

P1: The action of $\hat{P}$ is to deliberately distort the correct density *ρ*.

P2: The asymmetry only arises from the repeated coarse-graining every Δt in the *C*^+^ dynamics.

Conclusion: The coarse-grained asymmetry is an illusion.

Next I consider the anthropocentric objection. According to this objection, the coarse-grained asymmetry, in particular the coarse-grained entropy, is not an objective physical quantity, like energy or mass but rather is ‘agent-centric’. For example, Wigner and Jaynes have called entropy ‘anthropocentric’ (Jaynes [1965]). The terms ‘subjectivity’ and ‘anthropocentrism’ are used interchangeably in this debate. Denbigh and Denbigh ([1985]) helpfully distinguish two kinds of objectivity (and thereby of subjectivity). Objectivity_1_ is intersubjective agreement. Objectivity_2_ is stronger. It requires the phenomena in question to be independent of human cognition. In the debate about coarse-graining, intersubjective disagreement is not the issue. Rather it is the second kind of subjectivity (¬Objectivity_2_) that is at stake, which I earlier dubbed anthropocentrism.

The reason for this charge of anthropocentrism is as follows: In the case of the archetypal Gibbsian coarse-graining ${\hat{P}}_{cg}$ the size of the boxes is chosen by us. ’There are no laws of physics which determine the size of the [cells]’ (Denbigh and Denbigh [1985], p. 51), it is merely our preference that determines the choice. Furthermore, ‘the increase of entropy and the approach to equilibrium would thus apparently be a consequence of the fact that we shake up the probability density repeatedly in order to wash away all information about the past, while refusing a dynamical explanation for this procedure’ (Uffink [2010], p. 196). In addition, the partition is chosen by us: ‘the occurrence and direction of a temporal change of the entropy […] depends essentially on *our human choice* of the *size* of the finite equal cells of boxes into which we partition […] phase space’ (Grünbaum [1973], p. 647). The objection extends to all instances of $\hat{P}$; ‘a Zwanzig projection (describing generalized coarse-graining) can be arbitrarily chosen for convenience’ (Zeh [2007], p. 67)

Grünbaum ([1973]) points out that the charge of anthropocentrism here differs from the more general claim that scientific theories are human constructs. It seems that the Standard Model could describe the world, even if there were no (human) observers. Yet, according to the anthropocentric critique, this would not be the case for entropy, and the coarse-grained description.

Lying behind these objections is a particular justification of coarse-graining, the MI justification, to which I now turn.

### 4.2 Against the justification by measurement imprecision

In the literature, the most common justification for coarse-graining is that our measurements have limited precision: ‘The coarse-graining approach makes essential use of the observation that we only have access to measurements of finite resolution’ (Ridderbos [2002], p. 66). Thus, we can never locate a system precisely in phase space; we only know *p* and *q* to a certain degree of accuracy. The cells over which we average with the ${\hat{P}}_{cg}$ for the archetypal Gibbsian coarse-graining have a size that corresponds to ‘the limits of accuracy actually available to us’ (Tolman [1938], p. 167). Because we could never, *ex hypothesi*, measure the system accurately enough, we are unable to distinguish between the coarse and fine-grained distributions *ρ* and *ρ_r_*. Thus, according to this MI justification, the answer to choice is that we must pick the coarse-graining $\hat{P}$ that matches our observational capacities. For those coarse-grainings $\hat{P}$ whose selection is justified by the indistinguishability between *ρ* and *ρ_r_*, the MI justification also answers why (for those particular projections) coarse-graining at all is justified—because we cannot tell the difference.

Appealing to appearances originates from Gibbs’s ink analogy. While the ink drop’s volume is constant, it fibrillates throughout the water, and so it appears to us to be uniformly distributed. Our limited powers of observation cannot distinguish between the fibrillated case and the locally uniform distribution resulting from coarse-graining.

A similar argument arises in the Boltzmannian approach to SM, where phase space is partitioned into ‘macrostates’. Every microstate corresponds to one macrostate. A particular macrostate is defined by values of macrovariables, such as volume, temperature and pressure. These macrostates are sets of microstates that are ‘empirically indistinguishable’. Thus, an appeal is once again made to our observational capacities.^14^

The illusory and anthropocentric objections arise from this justification of coarse-graining (rather than coarse-graining itself). The claim that the coarse-grained asymmetry is illusory is bolstered by the MI justification, since it implies that if we were to be able to measure the system more precisely (in the idiom of Gibbs’s analogy to see the thin fibrillating tubes of ink rather than the smooth spreading) then the asymmetry would disappear. The coarse-grained asymmetry would thus be an illusion stemming from the imprecision of our measuring devices. The claim that the asymmetry is anthropocentric is also underwritten by the MI justification. If the coarse-grained *ρ* distribution is indistinguishable from the fine-grained *ρ* distribution to us and thus the choice of $\hat{P}$ depends our capabilities, then the asymmetry would be anthropocentric.

However, the MI justification is unsatisfactory. This is not (only) because it leads to the illusion and anthropocentric objections, but also, even on its own terms: it is both insufficient and unnecessary for justifying coarse-graining. (However, other purposes for which MI may be important will be briefly discussed in Sections 7 and 8.1).

The imprecision of our measurements is not a sufficient justification for implementing a coarse-graining projection $\hat{P}$, since choosing a projection that fits with the limits of observation will not always lead to autonomous irreversible dynamics of the type given by the ZZW framework: ‘Observability of the macroscopic variables is not sufficient […] It is conceivable (and occurs in practice) that a particular partition in terms of observable quantities does not lead to a Markov process’ (Uffink [2010], p. 196). That is, a coarse-graining could reflect our measurement precision but not lead to an example of useful dynamics—in particular, to autonomous *C*^+^ dynamics. Therefore, MI is not sufficient for answering choice.

Furthermore, appealing to MI is not necessary for explaining why we should choose any particular coarse-graining $\hat{P}$. If it were, we would in every case have to ascertain the imprecision of particular measuring devices and accordingly choose a coarse-graining $\hat{P}$. Yet, in Section 2, this is not how coarse-graining projections were chosen; and the details of particular measuring devices (or the resolution of our eyes) are in fact never used in constructing equations in the ZZW framework. It seems unlikely that advances in the science of microscopy will lead to different choices of $\hat{P}$.^15^

Thus appealing to the limited precision of our measurement devices is incapable of justifying the choice of coarse-graining projections (choice). The MI justification only answers at all in virtue of answering choice in particular cases, and thus its failure to answer to choice means that it automatically does not answer at all. With MI thus rebutted, I now outline my proposed alternative justification.

## 5 An Alternative Justification

Applying $\hat{P}$ throws away details. Why would throwing away details ever be a good move? One motivation for moving to the coarse-grained description is that modelling the evolution of *ρ* under the Liouvillean dynamics is computationally intractable, because solving the equations of motion for some 10^23^ particles is unfeasible.

Were this the only motivation for coarse-graining, one might be misled into believing that in an ideal world where we were equipped with a sufficiently powerful computer and the initial states of each of 10^23^ particles, the coarse-grained description would be dispensed with. Yet something would be lost, if upon receiving all the information and extraordinarily powerful computers, we ditched the discipline of SM. And this reveals a general point about the assumptions in SM: as I argue in Section 5.1, computational intractability is not the only motivation for such approximations and assumptions. In Section 5.1, I distinguish between Galilean idealization and abstraction, and then classify coarse-graining as abstraction to a higher level of description. This, plus the desideratum that the dynamics at this level be autonomous, allow me to justify coarse-graining. Then, in Section 5.2, I illustrate these ideas of abstraction and autonomy with the Game of Life.

### 5.1 Abstraction and autonomy

There are two reasons that such leaps in our computational capacity would not make SM ‘superfluous’. First, it is unclear in what sense solving some 10^23^ coupled equations would constitute an explanation of the behaviour of the gas.^16^ Second, a statistical mechanical system such as a gas exhibits ‘perfectly definite regularities in its behaviour’ (Tolman [1938], p. 2). Such regularities would be lost amongst the morass of detail at the fundamental (or lower) level. This difference in levels of description is particularly vivid in the case of coarse-graining; by moving to the lower-level Liouvillean dynamics, we not only lose explanatory power but also some very useful equations that determine transport coefficients and relaxation times.

At this point, we need to distinguish different strategies for simplifying scientific descriptions. This is a large topic and the words at issue—idealization, abstraction and approximation—are terms of art that different authors construe differently, but I will crudely categorize strategies as Galilean idealizations or abstractions. A Galilean idealization introduces ‘deliberate distortions’ (Frigg and Hartmann [2006]), familiar from the standard examples of frictionless planes and perfectly rational economic agents. A common way to think about such idealizations is by analogy to a perturbative series. The behaviour of the target system is veridically described by the full series, but a successful idealized description is akin to the first term of the series. Adding the higher-order terms renders the idealized description more accurate and furthermore, explains the success of the idealization even if these terms are not actually calculated (Batterman [2010], p. 17). Often Galilean idealizations are used in order to render a problem more tractable—and in an ideal world, we would remove the idealization (and so add all the terms of the series in)—and this would lead to a more accurate representation.^17^

In contrast, I take abstraction to be the omission, or throwing away, of certain pieces of information (Thomson-Jones [2005]; Knox [2016]). This corresponds to a broad category in the literature: Weisberg’s ([2007]) minimal modelling, Cartwright’s abstraction, and Aristotelian idealization (Frigg and Hartmann [2006]). This category involves ‘throwing away details, stripping away, keeping only the core causal factors’.^18^

Thus I claim that coarse-graining is not a Galilean idealization. If it were, there would be certain details those inclusion would improve the coarse-grained description. Yet, in the ZZW framework, this is not so. Indeed, we know exactly which details would need to be added to render a more complete description—the information about the irrelevant degrees of freedom that we threw away! But clearly if we were to add *ρ_ir_* back in, we would no longer have the coarse-grained, and useful, equations found in Section 2.

Instead, coarse-graining is abstraction. *ρ_r_* omits irrelevant information, which has been discarded by $\hat{P}$. For instance, in the archetypal Gibbsian case, the action of the coarse-graining projection ${\hat{P}}_{cg}$ is to omit exactly how the probability varies across the coarse-graining cell as only the probability of the entire cell is relevant: ‘how full the cell is, rather than how it is filled’. Some projections take a density in a given equivalence class to be an exemplar of that class (Wallace [unpublished], p. 9). In such cases, only the fact that the density is in the equivalence class is relevant, not which member of the class it is. In the case of ${\hat{P}}_{\mu }$, information about the correlations between particles is omitted.^19^

Thus *ρ_r_* is a new variable germane to this higher-level of description implicitly defined by a given $\hat{P}$, rather than a distorted replacement of *ρ*, which is how an idealization conception of coarse-graining would interpret *ρ_r_*. As *ρ_r_* forms part of a higher-level of description it need not be in tension with *ρ*, just as descriptions in biology need not be in tension with descriptions in psychology. *ρ_r_* is not an ‘idealized’ version of *ρ* containing false elements, as omission need not get in the way of telling a true causal story (Lewis [1986]; Strevens [2008]). Thus, coarse-graining at all is justified because it allows us to abstract to a higher-level of description. This is my proposed answer to Section 4’s at all.

$\hat{P}$ abstracts to a higher level of description. Yet we don’t just want to abstract to a higher level; we want a theory of the goings-on at this level. For example, suppose ${\hat{P}}_{cam}$ coarse-grains the position and mass distribution of people in Cambridge to the centre of mass of this population. The information about the masses and locations of individuals has been thrown away, leaving a more abstract description of the population. However, discussing the centre of mass of Cambridge’s population is not going to be useful, if the only way to find out how this centre of mass moves is to consider the movement of all the individuals and then re-average. If we cannot say anything about what is going on a higher level of description without invoking information from the lower level, then the higher level of description is not going to be useful.^20^

But not having to refer to the lower-level details in describing the goings-on at the higher level of description is precisely what the autonomy condition in the ZZW framework captures. Recall that the dynamics are autonomous if they were of the form $f(\rho_{r})$ rather $f(\rho_{r},\rho_{ir})$; the dynamics for the relevant degrees of freedom have no functional dependence on *ρ_ir_*. In other words, *ρ_ir_* is not a ‘difference maker’ for the evolution of *ρ_r_* (Woodward [2005]; Strevens [2008], Chapter 3). Note, however, that while the idea of different descriptions is contained in the concept of autonomy, no notion of hierarchy is implied. There could be different descriptions without one being ‘higher’ than another (see List and Pivato [2015], p. 150, Footnote 41). Thus the ‘higher-level’ aspect of this justification comes from taking $\hat{P}$ as abstracting from irrelevant details. The terminology of ‘relevant’ and ‘irrelevant’ degrees of freedom is highly appropriate; for if the dynamics weren’t autonomous then the so-called ‘irrelevant’ details would indeed be relevant.

Now, by taking this cue from the ZZW framework, it is clear what justifies the choice of any particular coarse-graining map. While any coarse-graining map can be used to find a pre-master equation, not every $\hat{P}$ will lead to coarse-grained irreversible dynamics. Only those coarse-grainings of a system that satisfy the two conditions (in Stage 3 of Section 2) will lead to autonomous dynamics.^21^ Thus, the choice of coarse-graining map is determined by whether it results in successful *C*^+^ dynamics. I agree that this criterion will not help physicists discover new, useful maps. The class of successful $\hat{P}$s will not look especially unified. But this is to be expected; each case requires details of the particular system at hand. Thus as Uffink ([2010], p.195) says: ‘it is ‘the art of the physicist’ to find the right choice, an art in which he or she succeeds in practice by a mixture of general principles and ingenuity, but where no general guidelines can be provided’.^22^

To summarize, this alternative justification answers Section 4’s two justificatory questions as follows:

Choice: The choice of a particular map is determined by the desideratum of finding autonomous dynamics.

At All: Applying a map $\hat{P}$ abstracts to a higher level of description.

### 5.2 An illustration: the Game of Life

The key ideas of autonomy and abstraction are vividly illustrated by Conway’s Game of Life—a standard example of the complexity science, and emergence, literature (see, for example, Bedau and Humphreys [2008], Chapters 8, 9, 11, 16, 17). The Game of Life is a cellular automaton that operates via a simple rule: at each time step, whether a cell of the grid is ‘on’ or ‘off’ is determined by how many of its eight neighbours are ‘on’. Despite the extreme simplicity of the dynamical rule, a rich variety of patterns can evolve in the grid. These stable shapes have characteristic movements and so are given vivid names: glider guns spawn gliders moving across the grid, eaters destroy other shapes they ‘encroach’ on, and puffer trains move across the grid leaving behind debris in their wake, to name but a few. While the sheer variety of the Game of Life cannot be easily conveyed in words (and is best appreciated by viewing a video of the evolution of a life grid), to give an idea of the complexity that can arise: the Universal Turing Machine has been constructed in the life grid (Poundstone [2013], p. 213).

When discussing the life grid, we can abstract to a higher level of description and, as done above, describe the goings-on in terms of the menagerie of ‘gliders’ and ‘blinkers’ rather than in terms of the cells. For example, the glider moves across the grid with velocity c/4, where *c* is the ‘speed of light’ (in the sense of being the ‘speed limit’—this maximum speed is one cell per unit time). This alternative description of gliders ‘has its own language, a transparent foreshortening of the tedious descriptions one could give at the physical level’ (Dennett [1991], p. 39). Discussing the gliders’ motion in this way is predictively successful. Furthermore, often these descriptions are autonomous: we need not keep referring back to the lower-level—that is, cell-level—details.^23^ But, of course, theoretically we could have calculated the evolution of the grid at the cell-level and then, at the end, abstracted to the higher-level, for example, glider-level, of description. Thus, as in the ZZW framework, there are two routes to predictions about later times; see Figure 2.

In both cases—ascending to the glider level of description from the cell level of description and ascending to a coarse-grained level of description (*ρ_r_*) from the fine-grained description (*ρ*)—new and surprising features emerge.^24^ In the Game of Life at the glider-level of description, there is ‘motion’. At the cell-level there is no motion. Likewise in SM. At the coarse-grained higher-level of description, many features are different. The coarse-grained probability density *ρ_r_*, the *C*^+^ dynamics, and the coarse-grained entropy *S_cg_* are very different from their fine-grained counterparts: the fine-grained distribution *ρ*, the microdynamics, *U*(*t*), and the fine-grained entropy, *S_fg_*. In the paradigmatic case of *N* particles in a box, the two descriptions give different answers regarding whether the dynamics is reversible or not, in particular, about whether the Gibbs entropy increases over a period of time or not.

Admittedly, there are differences. In the SM case, there are no patterns that can be ‘seen at a snapshot’. And because SM describes the evolution of probability densities there is no clear ontology at the higher-level description like Life’s menagerie.^25^ The pattern is the non-decreasing value of a particular quantity: the coarse-grained entropy, *S_cg_*. This is not a synchronic pattern but a dynamical pattern. Furthermore, unlike the Game of Life case this is not a visual pattern. However, patterns at higher levels of description need not be ‘visual patterns but, one might say, *intellectual* patterns’ that are ‘there for the picking up if only we are lucky or clever enough to hit on the right perspective’ (Dennett [1991], p. 41).

Yet, this in no way undermines its credentials as a pattern. One criterion for a higher-level pattern is predictive success, and betting that the coarse-grained entropy associated to an irreversible process will increase is a safe bet. Consequently, there ‘are macroscopic patterns running through those very microscopic interactions’ (O’Connor and Wong [2015], Section 1.4) in both the SM and Game of Life cases.

To summarize, the important consequence of coarse-graining, that is, of abstracting, is that autonomous dynamical patterns—structural features—once obscured by irrelevant details are revealed. Equipped with this alternative justification, I can now give a reply to the illusory objection in Section 6; and to the anthropocentric objection (in Section 7).

## 6 Reply to Illusory

Recall that two premises were required to establish the conclusion that the asymmetry is illusory. According to the illusory objector’s P1, coarse-graining distorts the correct density *ρ*. Furthermore, the coarse-grained asymmetry exists merely in virtue of the repeated coarse-graining every Δt in the *C*^+^ dynamics (P2). Thus, as the asymmetry is rooted in the infidelity of coarse-graining, it is illusory.

The immediate reply to illusory is surely—the irreversible equations of SM are empirically adequate. If the asymmetry were illusory then we could not expect such success. While this removes much of the force behind illusory, the illusory objector might deny our assumption of empirical adequacy. In any case, in this Section I argue that P2 is false and this refutes illusory. Furthermore, the considerations of Section 5 reveal that P1 is also false.

**Figure 3.**
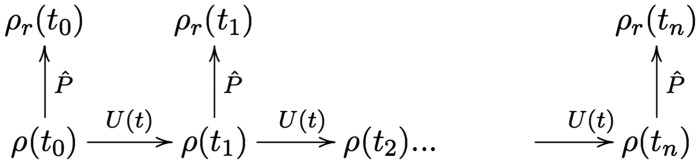
Route 1: To find the coarse-grained distribution *ρ_r_* at any given time, evolve the full-distribution under the microdynamics *U*(*t*) until this time and then apply the coarse-graining map $\hat{P}$.

Contra to P2, the asymmetry is not generated merely in virtue of the continual coarse-graining—provided that the forwards-compatibility condition is met, the asymmetry is robust with respect to the number of applications of $\hat{P}$. Even if we eschew the *C*^+^ dynamics, we could determine *ρ_r_* at particular times $t_{1},t_{n}$ by evolving *ρ* under the microdynamics then projecting up to *ρ_r_* at *t_n_*. Call this route 1 (as shown in Figure 3, a version of the forwards-compatibility diagram in Section 3.1). Taking route 1, we would still find that the coarse-grained variables, *ρ_r_* increase in entropy toward the future; $S(\rho_{r}(t_{0}))\leq S(\rho_{r}(t_{1}))\leq S(\rho_{r}(t_{2}))$. As such, we find an asymmetric pattern in *ρ_r_* without using the *C*^+^ dynamics. Thus, the asymmetry is not solely due to the repeated coarse-graining in the *C*^+^ dynamics and so, P2 is false.

P1 claims that the action of $\hat{P}$ is to deliberately distort the correct density. That is, coarse-graining is a Galilean idealization. On such a conception, *ρ_r_* and *ρ* are analogous to the first term and full series, respectively. According to illusory, neglecting these higher-order terms is the source of the asymmetry. However, Section 5 revealed that coarse-graining is not a Galilean idealization but rather an abstraction. *ρ_r_* is not a distorted replacement but a new variable germane to a higher-level of description. Consequently, P1 is false.

**Figure 4.**
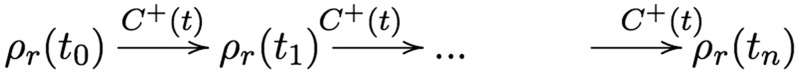
Route 2: To find the coarse-grained distribution *ρ_r_* at any given time, evolve *ρ_r_* under the *C*^+^ dynamics until that time. Recall the *C*^+^ dynamics is composed of applying *U* for Δt, applying $\hat{P}$, applying *U* for Δt…, where Δt is much smaller than $t_{1}-t_{0}$.

Ultimately, however, the falsity of P2 is key to rebutting illusory. The forwards-compatibility condition shows that the irrelevant degrees of freedom do not matter as they do not influence the evolution of the relevant degrees of freedom; they are not difference makers. As such, the coarse-grained asymmetry would be robust—even if coarse-graining were a Galilean idealization.

## 7 Reply to Anthropocentric

The anthropocentric objection is that no law determines the size of the cells and so we have a choice over which $\hat{P}$ to pick, and thus the coarse-grained quantities such as *S_cg_* are anthropocentric. The concern was that this marks SM out as a theory worryingly different from the rest of physics.

However, my proposed alternative justification (Section 5.1) claims that the choice of coarse-graining map depends upon whether it uncovers successful autonomous dynamics, not our limited capacities. Thus it is not that we have a choice over which $\hat{P}$ to pick (and consequently the resulting equations and *S_cg_* are ‘tainted’ by anthropocentrism). Rather it is a matter of whether *ρ_r_* and *ρ_ir_* dynamically decouple and ‘we are lucky or clever enough to hit on the perspective’—$\hat{P}$—that reveals the patterns that are ‘there for the picking up’ (Dennett [1991], p. 41). There is no freedom in the choice that makes it depend upon our cognition (in a way that differs from the rest of the scientific enterprise). Only for particular choices of $\hat{P}$ is there an autonomous dynamics —the choice needs to be ‘just right’ (Uffink [2010], p. 195). And this situation is not special. Like countless moves in physics—in particular, countless definitions of good variables—the use is justified by its success, where here ‘success’ means that autonomous dynamics are found.

Consequently, coarse-grained features need not be anthropocentric in a way different from other physical quantities and so in this matter, SM has the same status as any other scientific theory. Hence, coarse-graining does not lead to a specific anthropocentrism (which one might have been concerned would render SM incompatible with scientific realism).

However, as discussed in Section 5, different levels of description are useful for different purposes and what is deemed useful may be relative to our human interests. Here our measuring capacities and imprecision are certainly relevant. Were we the size of a Maxwell demon and endowed with an ability to manipulate gas molecules, violations of the second law of thermodynamics might be expected. From their microscopic perspective, the second law might not seem like an obvious regularity in nature.

In addition, which patterns are uncovered might depend upon our limited human capacities—whether we can ‘hit on the right perspective’. For instance, there may be regularities in the movement of the centre of mass of Cambridge’s population, but our cognitive abilities may make us unable to pick up these patterns. Which variables we find useful depends on which variables we can access, that is, measure and manipulate. Thus, our measuring capacities will clearly influence the construction and confirmation of our scientific theories. But—crucially—the details of our measuring limitations are not needed to justify coarse-graining in SM.

The above considerations highlight a potential general anthropocentrism. Our scientific theories may be irrevocably entwined with our cognitive abilities and pragmatic interests. But this is not the return of the earlier anthropocentric objection, which was specific, namely, that the coarse-grained features are anthropocentric in a way that differs from the other putative physical quantities. The alternative justification shows that coarse-graining need not mark out SM as subjective and so different from other theories, but this conclusion is nonetheless compatible with scientific theories in general containing some element of anthropocentrism.

## 8 The Wider Landscape: Concluding Remarks

In Section 4, one of the concerns about coarse-graining was whether the coarse-grained asymmetry is ‘real’ or not. Recall that Davies claims that this was ‘a matter of philosophy’, and indeed, in Section 8.1, I explain why this is so—briefly, whether the asymmetry is real or not depends on one’s views about inter-theoretic relations. Then in Section 8.2, I consider what my proposed justification reveals about the nature of irreversibility in SM.

### 8.1 Inter-theoretic relations

To some extent, the ZZW framework provides a case study in inter-theoretic relations; SM is a distinct, higher-level theory from either of CM or QM. In the wider literature on inter-theoretic relations, one key issue is the nature of the connections between the different levels. For instance, biology and psychology could be disunified descriptions operating at different levels of generality; in addition to not being ‘reducible-in-practice’, they could even be not reducible-in-principle (Bedau and Humphreys [2008], p. 215). That is, there may be disunity between the psychological and biological levels of description. Cartwright ([1999]), for example, advocates such a patchwork view of the scientific enterprise.

Different philosophical accounts of reduction make different requirements on the notion, and some are more stringent than others. (For instance, there is debate about whether any bridge laws invoked by the reduction must ensure the lower-level theory explains the higher-level theory). I will leave aside the details of different accounts of reduction, since I think that independently of any given account of reduction, this is a case of reduction-in-practice. After all, the ZZW framework allows us to construct the equations of one theory (SM) from another (CM or QM).

But there is a further issue concerning inter-theoretic relations: what attitude should one have to the higher-level entities, realism or instrumentalism? Hence, as Davies says, whether one believes the coarse-grained asymmetry is ‘real’ is a matter of philosophy; it depends on your prior philosophical convictions about higher-level entities in the special sciences.

Furthermore, such philosophical convictions may also have a general impact on one’s views about the nature of the asymmetry. Had the MI justification been the best justification of coarse-graining, then the coarse-grained asymmetry would have been revealed to be inescapably subjective or anthropocentric.

While I hope to have established (in Sections 6 and 7) that one is not compelled to consider the asymmetry to be anthropocentric, motivated by general themes in inter-theoretic relations, one might still want to conclude that it is, in fact, anthropocentric.

For example, an instrumentalist about higher-level theories might maintain that the instrumental value of these descriptions is inextricably bound up with our measuring and cognitive capacities and thus, all higher-level entities are anthropocentric. The key message of this article is that the justification of coarse-graining need not mark SM out from other scientific theories for that discussion (as we saw in Section 7).

Next, there is a final philosophical issue about the nature of the coarse-grained asymmetry to discuss: its emergent nature.

### 8.2 The nature of irreversibility

Finally, I turn to irreversibility. As a foil for this discussion, I choose a passage from (Sklar [1993], p. 217), which puts very well a general doubt, namely, whether a strategy such as the one outlined in this article, can really succeed in reconciling the time-symmetry of micro-processes with the asymmetry of macro-processes: ‘Do the procedures for deriving kinetic equations and the approach to equilibrium really generate *fundamentally* time-asymmetric results?’ (emphasis added).

However, contra to Sklar’s phrasing, the ZZW construction method does not generate a fundamental time-asymmetry. The coarse-grained asymmetry is a feature of a higher-level description. Higher-level descriptions can have features that differ substantially from the lower-level descriptions (without there being a contradiction). Often these features are described as ‘emergent’.

‘Emergence’ is a murky word and is used in many different ways (for a survey, see Silberstein [2002]). Very roughly, emergent entities or processes ‘arise’ out of more fundamental entities or processes and yet have ‘distinctive’ features in their own right. It is contentious what the ‘distinctive’ features are; proposals in the literature include ‘novelty’ (Butterfield [2011], p. 1065) and being ‘unexpected’ (Chalmers [2006], p. 244).^26^ Furthermore, how substantively a phrase such as ‘in their own right’ must be read also varies across authors—some maintain that emergence is the failure of reduction while others (for example, Butterfield [2011]) deny this.

The menagerie of the Game of Life, such as gliders and blinkers, are often cited as key examples of emergent entities that have certain emergent properties and evolve under certain emergent processes (Bedau and Humphreys [2008]).

The sense in which I use ‘emergent’ is mild; it is merely that there is ‘novel and robust behaviour with respect to some comparison class’ (Butterfield [2011], p. 1065). (Butterfield’s account is especially apt for this case, since he shows his definition to be compatible with (Nagelian) inter-theoretic reduction, and as discussed above, the ZZW construction is a case of reduction).

Of course, as mentioned above, there are many accounts of emergence that one could favour. An alternative account that might seem apt here is Wilson’s ([2010]). Her key idea is that some phenomena are ‘weakly ontologically emergent from physical phenomena’ ([2010], p. 280) when some degrees of freedom are eliminated.

Note that eliminating functional dependence of one set of degrees of freedom from another was exactly the autonomy condition of the ZZW framework. Furthermore, her accounts fits well with the general topic of abstraction and talk of levels of description (such as that made precise by List and Pivato [2015]). However, Wilson’s focus is on weakly emergent entities and as mentioned at the end of Section 5.2, one of the disanalogies with the Game of life is that is unclear in our case what the candidate emergent entities would be. Moreover, Wilson’s aim is defend non-reductive physicalism, which is contentious. Thus, I will not pursue Wilson’s account further here. Instead, I submit that the broad gist of Butterfield’s account captures the main intuition common to all accounts of ‘emergent phenomena’: robust, because a putative case of emergence must not be too flimsy in order to count as a *bona fide* phenomenon, and novel in order to earn the name ‘emergent’.

Thus, my response to Sklar’s concerns above is as follows: The irreversibility generated by these methods is not fundamental but emergent. Irreversibility emerges when one abstracts from the fine-grained level of description to the coarse-grained level of description by applying a $\hat{P}$ that leads to autonomous dynamics.

Note finally that this mild conclusion that the coarse-grained asymmetry is weakly emergent is not ‘toothless’. It is in direct opposition to Prigogine and Stengers ([1984], p. 285) who claim: ‘Irreversibility is either true on all levels or on none: it cannot emerge as if out of nothing, on going from one level to another’. While the lower-level dynamics is reversible, the coarse-grained dynamics at the higher level of description is irreversible. True, this emergent irreversibility does not arise ‘as if out of nothing’. Time-asymmetric assumptions were required when constructing the *C*^+^ dynamics (and when ruling out the *C*^*−*^ dynamics) in Section 2. But this is to be expected; if no asymmetry is put in, then we cannot expect asymmetry out.^27^

To sum up: The ZZW framework constructs the irreversible equations of SM from the underlying reversible microdynamics thus reconciling the higher-level asymmetry with the lower-level symmetry. The procedure of coarse-graining—key to this reconciliation but thought to be suspicious by many—was justified provided that coarse-graining allows us to abstract to a higher-level autonomous description (in a manner illustrated by the Game of Life). I used my justification of coarse-graining to show that the coarse-grained asymmetry is neither illusory nor anthropocentric; instead, it is weakly emergent.

## Footnotes

^1^ Well, almost. The relevant symmetry is the CPT-invariance, but the failure of TR-invariance in subatomic physics doesn’t underpin the asymmetries discussed here. See, for example, (Roberts [2013], [2017]) for the subtleties of TRI.

^2^ Throughout this article, I leave the interpretation of such probability densities open; but admittedly, their connection to the behaviour of individual systems is an urgent issue in the philosophy of SM (see, for example, Sklar [1993], Chapter 3).

^3^ $\hat{P}$ is idempotent: ${\hat{P}}^{2}=\hat{P}$. $\hat{P}$ is usually linear and time-independent and so commutes with $\frac{\partial }{\partial t}$.

^4^ The condition for an equation to be autonomous, familiar from mathematics, is that ‘*t* does not occur explicitly in the equation, as in $\frac{dy}{dt}=f(y)$’ (Robinson [2004], p. 13). This is required so that $\frac{\partial \rho_{r}(t)}{\partial t}$ has no ‘covert dependence’ on *ρ_ir_*.

^5^ This is a sufficient but not necessary condition for this term to vanish; the action of $\hat{P}Le^{-it(1-\hat{P})L}$ on a non-zero $\rho_{ir}(0)$ could also be such that the term disappears.

^6^ This general assumption of e term disappears.ion for of course used much more widely than just in the ZZW framework. For example, in (Reif [2009], Chapter 14), the derivation of the Boltzmann equation requires a similar assumption: that $f(\vec{r},\vec{v},t)$ does not vary appreciably during time intervals of the order of the collision time, nor over spatial intervals of the order of intermolecular forces. I now offer an intuitive explanation of the general situation, by extending Zeh’s discussion using a metaphor of a forest. Within the irrelevant information, Zeh ([2007], p. 65) distinguishes the ‘doorway’ from ‘deep states’, which are in different ‘channels’. So there are three ‘channels’: (A) ‘relevant’, (B) ‘doorway’, (C) ‘deep’—and these are analogous to (A) a clearing in a wood, (B) the sunny woodland surrounding the clearing, (C) the dark woods. Zeh gives the following example: (A) is a one-particle marginal density, (B) encodes two-particle correlations and (C) encodes three-or-more particle correlations (cf. the BBGKY hierarchy). Now, the non-Markovian term in the pre-master equations gives the contributions to $\frac{\partial \rho_{r}(t)}{\partial t}$ at *t* from the part of *ρ_r_* that became irrelevant at t−t′ and remained irrelevant until time *t*: at which point it becomes relevant again. This ‘information becoming irrelevant’ is like people in the clearing (A) wandering into the sunny woodland (B). Thus, the relaxation time *τ* is the time taken for the people who arrived in the sunny woodland (B) to wander either back to the clearing (A) or into the dark woods (C). The key assumption is that once in the dark woods (C), no one can find their way back to the clearing (A) again. In less picturesque terms: the three-or-more particle correlations are not dynamically relevant for the one-particle marginal density. This metaphor also encompasses the famous recurrence theorem. If you wander around a (finite) woodland for an incredibly long (that is, recurrence) time, you will eventually find your way back to the clearing. As I will discuss in Section 3.1, on recurrence timescales the ‘deeply’ irrelevant states (C), for example, three-or-more particle correlations, become relevant (A) again.

^7^ More accurately, the spin’s velocities are unaltered, but the order of the spins is altered by reflection in the *x*-*z* plane. However, ‘the grain of truth in the standard story is that a reversal of the ordering with unaltered velocities is in a sense ‘isomorphic’ to a velocity reversal with unaltered ordering’ (Frigg [2010], p. 64)

^8^ Blatt ([1959], p. 749) concludes that ‘it is not permissible to base fundamental arguments in statistical mechanics on coarse-graining’.

^9^ Lavis ([2004], p. 686) further defends coarse-graining.

^10^ One might object that this definition is vague. Instead, consider this ‘simplicity’ condition as an overarching condition to capture what is similar across those densities that satisfy the initial state assumption for different $\hat{P}$s. A given *ρ* satisfying the initial state assumption at *t*_0_ will ensure that—with respect to a given $\hat{P}$ and thus a given definition of ‘irrelevant’—*ρ* is simple at *t*_0_. But of course there are many densities that count as simple in some sense, but not in the respect required for the initial state assumption ($\rho_{ir}(t_{0})=0$) for a particular $\hat{P}$.

^11^ Wallace points out that it would be excessive to apply it more than once; the microdynamics are deterministic and so fixing *ρ* at one time fixes *ρ* for all times.

^12^ The plausibility of such initial conditions on probabilities densities will depend on one’s interpretation of probability in SM. Simplicity fits especially well with a Jaynesian account: Jaynesians interpret *ρ* as encoding our ignorance of the system. If all we know is the system’s macrostate, then claiming that *ρ* is uniform across this state ensures that *ρ* is simple.

^13^ The difference between *t*_0_ for Options 1 and 2 is dramatic: 13.7 billion years. One might think that this should lead to equally dramatic differences in the constructed equations, as *t*_0_ appears in the premaster equation. And thus one might hope to adjudicate between Options 1 and 2 on these empirical grounds. But the key physical insight behind the Markovian approximation explains why despite the dramatic difference between *t*_0_ for Options 1 and 2, there need be an accompanying dramatic empirical difference, as follows: Provided that the Markovian approximation holds good and—as is uncontroversial—the recurrence time is much greater than 13.7 million years, if we apply $\rho_{ir}=0$ at the beginning of the universe, there will not be 13.7 billion years’ worth of ‘irrelevant information’ (for example, correlations encoded in *ρ_ir_*) that is liable to be about to become dynamically relevant for *ρ_r_* (and so causing empirical differences between Option 1 or 2). The only potential difference will be the information *τ* seconds ago. (For more details, see Zeh [2007], p. 64.)

^14^ But the Boltzmannian partition is not necessarily the same as the Gibbsian cells.

^15^ My rebuttal of the necessity of the MI justification takes its proponents at their word. But perhaps this is uncharitable, for in reality, typical discussions of the construction of autonomous equations are often schematic—they merely assume there is such a projector that satisfies the required assumptions, without a detailed demonstrations that the projector does indeed fulfil these assumptions. As I have not investigated such demonstrations, it is an open question whether for that project—rather than the construction of autonomous dynamics—measurement imprecision is necessary.

^16^ Some might argue that while the solution of the 10^23^ equations might not be the best explanation, it is nonetheless an explanation. However, the details of the vast debate about explanation are not needed for what follows.

^17^ In Weisberg’s ([2007], p. 642) terminology, the ‘representation ideal’ would be to remove the idealization.

^18^ Of course, there are other categorizations—for example, McMullin ([1985]) has six types of idealization. Norton ([2012]) discusses approximation and idealization in a different sense. Notably, different $\hat{P}$s might be (sub)-categorized differently according to a more fine-grained classification. However, all that matters here is that coarse-graining is not a Galilean idealization.

^19^ Of course, adding in some correlations (that is, adding in the third tier of the BBGKY hierarchy) may lead to a more empirically successful autonomous equation than the autonomous equation describing the evolution of the one-particle marginal density. Indeed, for example, doing so can provide corrections to the Boltzmann equation. But of course, we wouldn’t want to add in all the correlations in the BBGKY hierarchy—in the limit, doing so would take us back to the reversible dynamics. And indeed, truncation at first or second equations in the hierarchy is the key benefit of the BBGKY approach—it is useful because we can get away with only considering the lower hierarchy.

^20^ List and Pivato ([2015], p. 135) go further. In their framework, the lower-level language is by definition unavailable at the higher level.

^21^ Autonomy in the sense of ‘not referring to *ρ_ir_*’ is achieved by the initial state assumption. But for autonomy in the sense of not depending at all on *t*, the Markovian approximation needs to be satisfied.

^22^ Of course, in individual cases, there will be the further explanatory project of showing that the two required assumptions are satisfied by a chosen $\hat{P}$—and this will give us further insight into why in these particular cases autonomous dynamics are possible, that is, why our desideratum is fulfilled. But as a general answer to (Choice)—the only rationale for picking any $\hat{P}$ is that it leads to an autonomous dynamics.

^23^ This is akin to autonomy in the SM case, although not literally as there are no differential equations.

^24^ Of course, in both cases, finding these features will depend sensitively on how the higher-level variables are defined, that is, on how we abstract; see (Knox [2016], p. 45).

^25^ Note, also, that Life differs in another way: the patterns in Life are noise-intolerant—‘debris’ can easily destroy the menagerie.

^26^ Both of these examples are definitions of ‘weak emergence’ (a use of the word ‘emergence’ popular with scientists and philosophers of physics) as opposed to the philosopher’s ‘strong emergence’, which is a logically stronger notion. Although authors vary about exactly what the distinction between weak and strong emergence is, the idea is that this stronger sense implies a lack of reduction or supervenience of the emergent phenomenon on the lower level. See (Chalmers [2006]) for more detail on the weak/strong distinction.

^27^ Since the time-asymmetry is not fundamental and was ‘put in by hand’ (as discussed earlier), this project won’t satisfy those seeking to locate the source of time-asymmetry. To the extent that this project answers that question, it claims the asymmetry arises because of particular initial conditions (the initial state assumption). Some want an explanation of such initial conditions, especially when in the guise of a past hypothesis (see Callender [2004]; Price [2004]), especially since such initial constraints seem *ad hoc* or unnatural from ‘the mechanical world-view’ (Sklar [1993], p. 368). Moreover, there is a debate over whether such an initial state is a law or a ‘de facto’ condition (Reichenbach [1991]; Grünbaum [1973]; Sklar [1993], p. 370). Some, such as Krylov ([1979]), are unhappy with the centrality of such initial conditions in explaining irreversibility. But I believe my conclusion about the emergent nature of the asymmetry helps us to see which explanatory projects are likely to be fruitful. In particular, my conclusion eases the worry that the initial conditions required do not look especially natural nor form a unified class. Because these higher-level patterns are weakly emergent, they are unexpected from the lower-level mechanical perspective. Thus, the moves required at the lower-level in constructing SM equations may often look unnatural; otherwise, the higher-level pattern would have been expected.
